# Metastatic High-Grade Serous Ovarian Carcinoma Presenting as a Temporal Lobe Glioblastoma Mimic: A Diagnostic Pitfall

**DOI:** 10.7759/cureus.104816

**Published:** 2026-03-07

**Authors:** Machina Sravya, Inuganti Venkata Renuka, Vaddatti Tejeswini, Amulya Boddapati, Nikitha Katragadda

**Affiliations:** 1 Department of Pathology, NRI Academy of Medical Sciences, Guntur, IND

**Keywords:** brain metastasis, glioblastoma mimic, high-grade serous ovarian carcinoma, immunohistochemistry, ovarian carcinoma

## Abstract

High-grade serous ovarian carcinoma (HGSOC) is the most common subtype of ovarian cancer, known for its aggressive nature and unique pattern of metastasis. Brain metastases from ovarian carcinoma are uncommon, with HGSOC rarely presenting with multifocal lesions that radiographically mimic the multi-centric growth pattern of a primary glioblastoma, particularly following a prolonged period of clinical remission, leading to diagnostic ambiguity. We report the case of a 69-year-old woman with a prior history of HGSOC who presented with new-onset cognitive and language deficits. Neuroimaging indicated a prominent temporal lobe mass radiologically suggestive of glioblastoma. Histopathological examination and immunohistochemistry demonstrated features consistent with metastatic HGSOC, showing strong paired box gene 8 (PAX8) positivity and absence of glial markers. This case highlights the importance of considering a range of differential diagnoses for intracranial lesions in patients with a history of malignancy by using a multidisciplinary approach to interpret clinical, radiologic, and histopathologic data, while emphasizing the crucial role of immunohistochemistry in distinguishing metastatic disease from primary brain tumors.

## Introduction

While brain metastases are a frequent complication of systemic malignancies, central nervous system (CNS) involvement in ovarian cancer remains rare, reported in only 0.49% to 6.1% of cases, with an estimated occurrence of 1.34% [1]. High-grade serous ovarian carcinoma (HGSOC) is an aggressive epithelial malignancy arising from Müllerian epithelium and represents the most common histological subtype of ovarian cancer. It typically spreads via trans-coelomic dissemination and lymphatic routes, making CNS involvement uncommon [2]. When present, brain metastases usually occur late in the disease course and are often multiple [3]. Glioblastoma is the most common primary malignant brain tumor in adults and frequently presents as a heterogeneously enhancing mass with necrosis and surrounding edema. Distinguishing solitary brain metastases mimicking primary glial tumors on imaging poses a significant diagnostic challenge, especially after a prolonged interval, but it is critical, as treatment strategies differ substantially.

We describe a rare case of metastatic HGSOC presenting as a temporal lobe mass radiologically resembling glioblastoma, emphasizing the importance of histopathological and immunohistochemical evaluation in establishing the correct diagnosis.

## Case presentation

A 69-year-old woman of Indian origin presented to a tertiary care hospital with complaints of memory disturbances for three weeks, difficulty in calculation for 10-20 days, and speech abnormalities characterized by impaired comprehension and reduced fluency.

She had a known history of HGSOC diagnosed in 2022 and received neoadjuvant chemotherapy with paclitaxel and carboplatin for four cycles between November 2022 and February 2023. She subsequently underwent interval debulking surgery on March 21st, 2023, including total abdominal hysterectomy, bilateral salpingo-oopherectomy, bilateral pelvic node dissection, and omentectomy. The International Federation of Gynecology and Obstetrics (FIGO) stage was not documented in the accessible records. Final pathological staging following neoadjuvant therapy was ypT2 ypN0 (y-post-treatment staging, p-pathological classification). Post-operatively, she completed two additional cycles of paclitaxel and carboplatin, concluding systemic therapy in June 2023. She had a disease-free interval of approximately 28 months with no systemic disease recurrence before presenting with neurological symptoms in late 2025. There was no history of seizures, headache, vomiting, or gait disturbances.

Clinical examination

On examination, the patient was conscious and oriented, with a Glasgow Coma Scale score of 15/15 (E4V5M6). Neurological evaluation revealed mild cognitive impairment involving recent memory and language functions, consistent with left temporal lobe involvement. Cranial nerve examination and motor system evaluation were normal, and there were no signs of raised intracranial pressure.

Investigations

**Figure 1:**
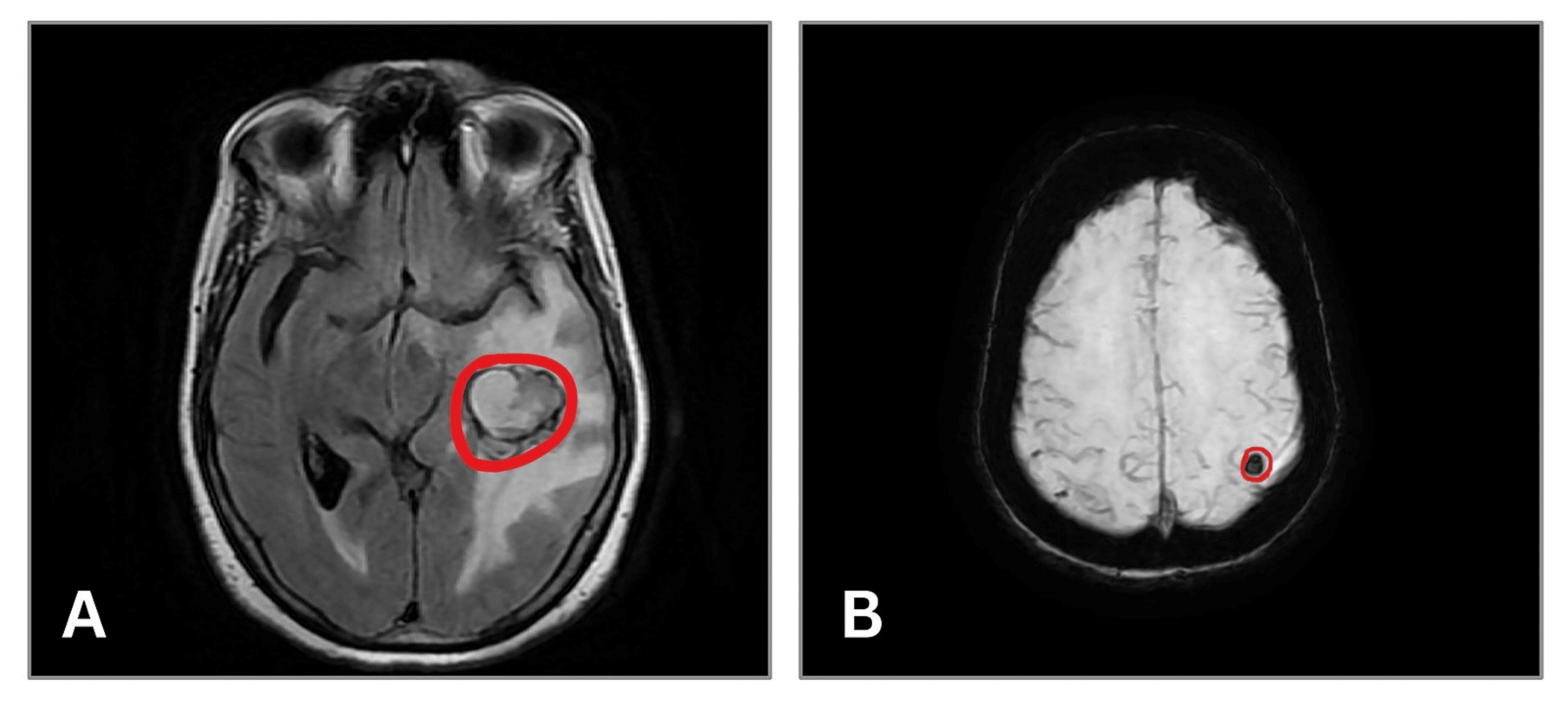
Magnetic resonance imaging (MRI) of the brain demonstrating intracranial lesions (A)  Axial diffusion-weighted image demonstrating a well-defined lobulated solid-cystic lesion in the left temporal lobe with diffusion restriction and surrounding perilesional edema (circled). (B) Axial image demonstrating nodular lesion in the left parietal lobe showing blooming on susceptibility-weighted imaging (SWI) with disproportionate perilesional edema (circled).

Magnetic resonance imaging (MRI) of the brain revealed a well-defined lobulated solid-cystic mass in the left temporal lobe measuring 2.8 × 2.7 × 2.0 cm with diffusion restriction and significant perilesional edema causing a midline shift of 7.5 mm. A small enhancing nodule measuring 6.6 × 5.5 mm was also identified in the left high parietal region (Figure 1). These imaging findings were suggestive of a high-grade glioma [4].

Differential diagnosis

Based on clinical and radiological findings, the differential diagnoses included a high-grade glioma, a metastatic brain tumor, and primary CNS lymphoma.

Histopathology and immunohistochemistry

**Figure 2:**
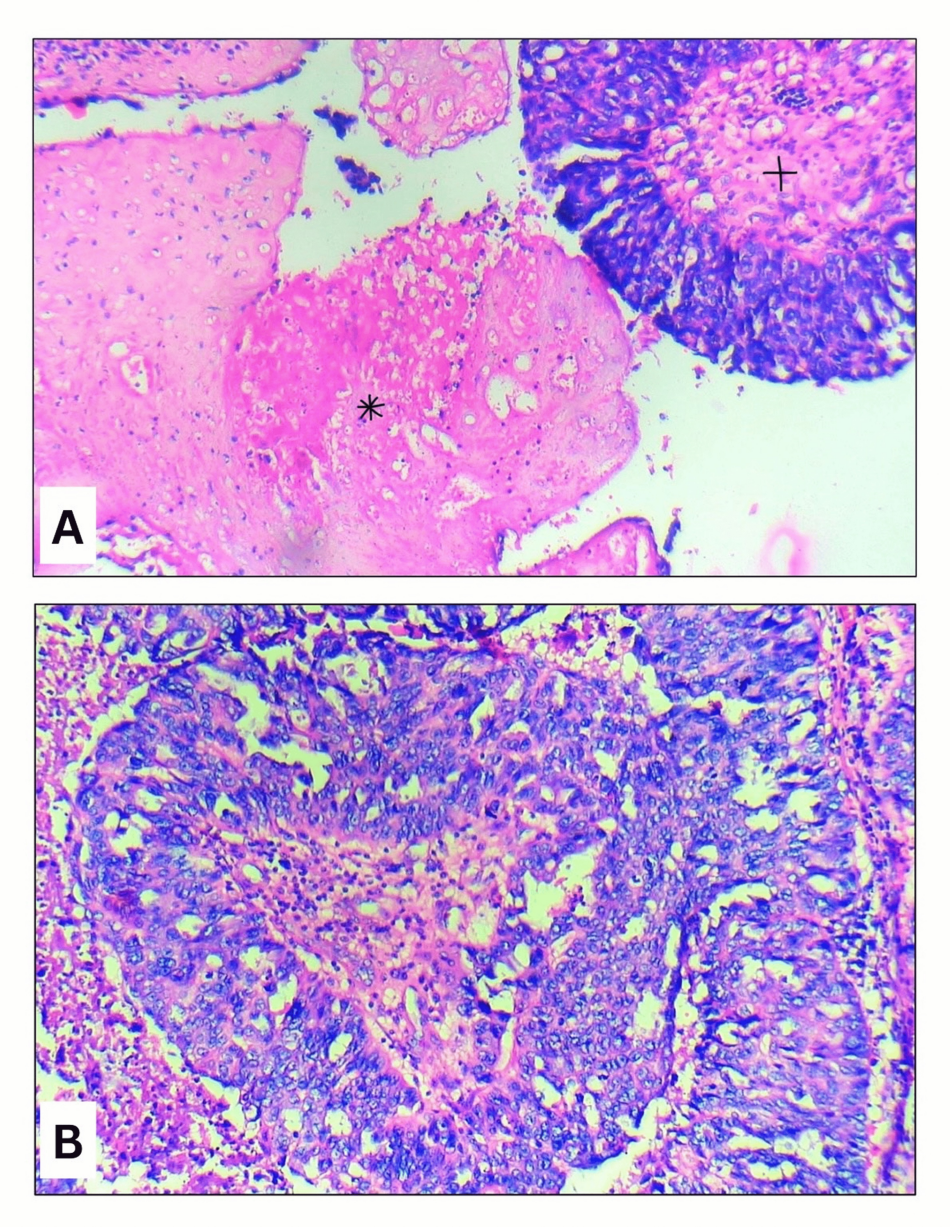
H&E stained photomicrographs (A) Low-power view of the tumor-brain interface showing glial tissue (*) and ovarian serous carcinoma (+) components (H&E, x100). (B) High-power view of the tumor showing cells arranged in papillary and cribriform patterns with marked nuclear pleomorphism, vesicular nuclei, and prominent nucleoli (H&E, x400).

**Figure 3:**
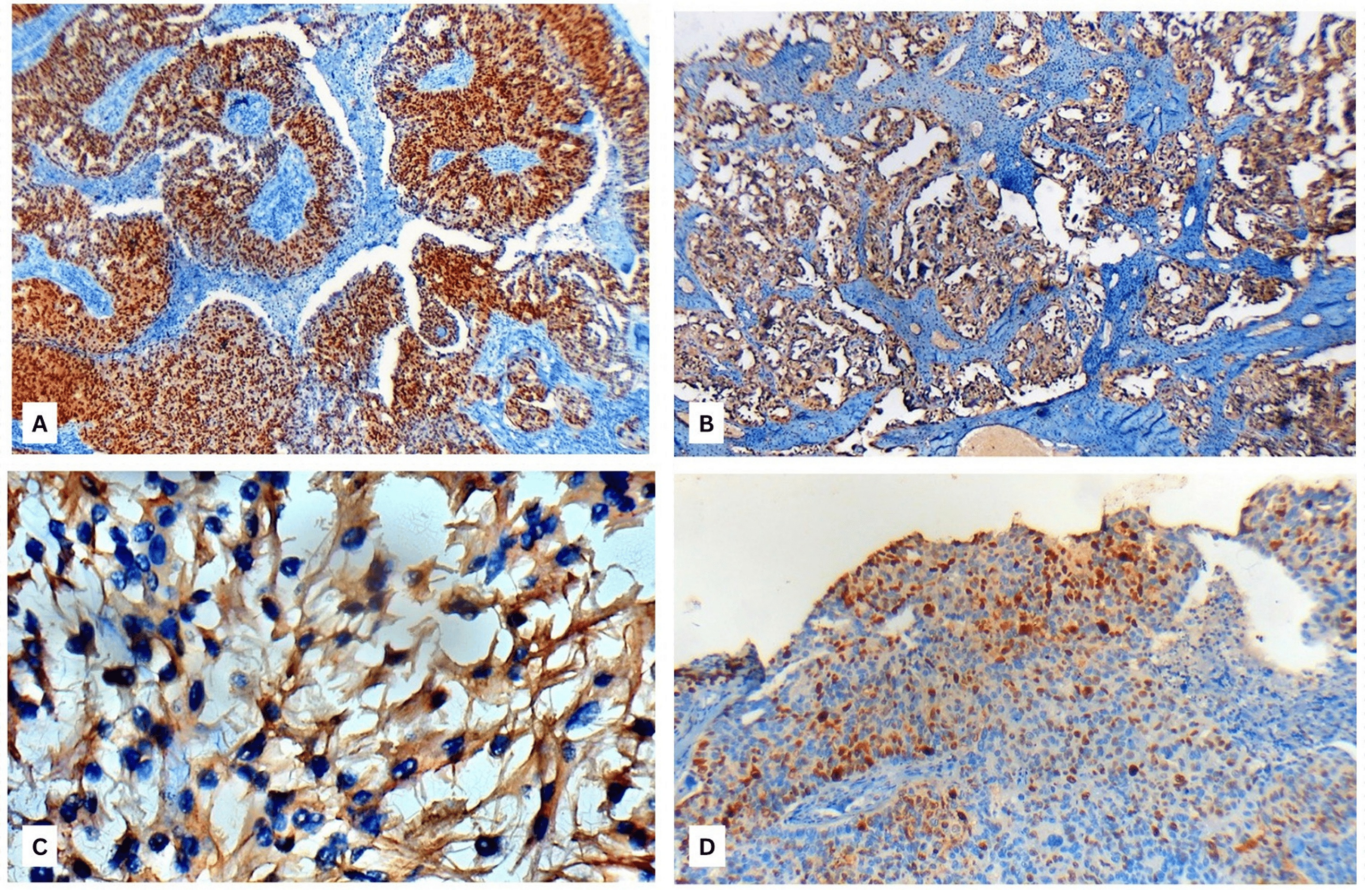
Immunohistochemical profile of the tumor (A) Strong nuclear positivity for PAX8 in tumor cells (IHC, x100). (B) Diffuse strong nuclear and cytoplasmic positivity for p16 in tumor cells (IHC, x100). (C) Absence of glial fibrillary acidic protein (GFAP) expression in tumor cells (IHC, x400). (D) High Ki-67 labelling index of approximately 40% (IHC, x100).

The specimen received consisted of tissue from a left temporal lobe lesion obtained via gross total excision. Histopathological examination revealed tumor cells arranged in papillary and cribriform patterns with marked nuclear pleomorphism, vesicular nuclei, prominent nucleoli, and moderate eosinophilic cytoplasm. Areas of focal necrosis, hemosiderin-laden macrophages, and inflammatory infiltrates were also observed (Figure 2). Immunohistochemical analysis demonstrated strong paired box gene 8 (PAX8) and p16 (cyclin-dependent kinase inhibitor 2A) positivity, a high Ki-67 (MKI67) proliferation index with absence of expression of glial fibrillary acidic protein (GFAP), Napsin A and p63 (Figure 3).

Although broader Müllerian panels including WT1 and CK7 are often recommended in the literature, in this case, the combination of strong PAX8 positivity, characteristic morphology, negative glial markers, and a known history of HGSOC was considered sufficient for diagnosis; therefore, additional markers were not pursued. The profile supported a diagnosis of metastatic HGSOC of Müllerian origin and excluded a primary glial neoplasm (Table 1)*.* The typical radiologic patterns and immunohistochemical profile described in the literature are summarized in Table 2 for better understanding of the diagnostic overlap with the present case.

**Table 1:** Immunohistochemical profile of the intracranial tumor Immunoprofile showing strong PAX8 and p16 positivity with absence of glial markers, supporting a diagnosis of metastatic high-grade serous ovarian carcinoma of Müllerian origin.

Marker	Result	Staining Pattern	Diagnostic Significance
PAX8	Positive	Strong nuclear positivity	Supports Müllerian ​origin; consistent with metastatic high-grade serous ovarian carcinoma.
p16	Positive	Diffuse strong nuclear and cytoplasmic positivity	Seen in high-grade serous ovarian carcinoma; reflects underlying TP53 alteration
GFAP	Negative	No tumor cell staining	Excludes primary glial neoplasm (glioblastoma)
Napsin A	Negative	No tumor cell staining	Helps exclude pulmonary/renal origin
p63	Negative	No nuclear staining	Excludes squamous cell carcinoma
Ki-67	~40%	High nuclear labeling index	Indicates high rate of proliferation

**Table 2:** Comparative summary of typical radiologic, histopathologic and immunohistochemical features of glioblastoma and metastatic high-grade serous ovarian carcinoma* Key differences between glioblastoma and metastatic high-grade serous ovarian cancer for diagnosing lesions. *This table summarizes characteristic features reported in the literature [5-7] and does not represent the findings of the present case. HGSOC: High-grade serous ovarian carcinoma

Feature	Glioblastoma (GBM)	Metastatic HGSOC
MRI Appearance	Heterogeneous ring-enhancing lesion, crosses midline usually (butterfly glioma).	Heterogeneous necrotic soft tissue masses. Ring-enhancement, often at grey-white junction.
Growth Pattern	Infiltrative	Well-circumscribed/expansile
Typical IHC Profile	GFAP (+), S100 (+)	PAX8 (+), WT1 (+), CA-125 (+)

Management and follow-up

In view of the symptomatic dominant cranial lesion, the patient underwent left temporal craniotomy with neuronavigation-guided gross total excision on November 1, 2025. Histopathologic examination confirmed metastatic HGSOC. Post-operatively, she received stereotactic radiosurgery (SRS) as CNS-directed adjuvant therapy for residual intracranial disease. At approximately four months of follow-up, the patient remains clinically stable without new neurologic deficits. No evidence of extracranial disease has been identified to date.

Because of the rarity of these patients, the optimal treatment for brain metastases is currently ill-defined [8]. It should be noted that the best survival results for brain metastases from ovarian cancer have been obtained with trimodal therapy (radiotherapy, surgery, and chemotherapy), while monotherapy is associated with poor survival [9]. She remains under periodic clinical and radiological follow-up to assess therapeutic response and disease progression.

## Discussion

Most of the reported cases of brain metastasis from ovarian carcinoma stated that patients were in their fifth to seventh decades. Pakneshan et al. reported a median age of 54.3 at the time of CNS involvement in their systematic review [10]. Our patient, aged 69 years, falls at the upper end of the spectrum, highlighting that delayed metastasis can occur even in elderly patients who have undergone definitive treatment. In a retrospective analysis by Wang et al., the most frequent metastatic sites were the cerebellum and brainstem. In our case, the patient presented with a dominant lesion in the left temporal lobe with an additional satellite nodule in the parietal region, the incidence of which is reported to be 9.3% and 11.6%, respectively, suggesting that it is a less common metastatic distribution compared to the most frequent sites reported in the above-stated study [11]. Neurological symptoms in reported cases commonly include headache, seizures, fatigue, and signs of raised intracranial pressure [12]. In contrast to this, our patient primarily presented with memory disturbances, calculation difficulty, and speech abnormalities with no features of raised intracranial pressure. This contributed to the impression of the lesion being a primary glioma.

Metastatic ovarian carcinoma lesions usually show ring enhancement, hemorrhage, and surrounding edema. Some authors have noted that a solitary metastasis may closely mimic glioblastoma. In our case, the lesion had solid-cystic architecture, diffusion restriction, susceptibility blooming, and extensive perilesional edema with midline shift, which are classical features of glioblastoma [13]. This appearance on imaging favored a primary glial neoplasm, making this a notable diagnostic pitfall. Our case showed papillary and cribriform growth patterns with marked nuclear pleomorphism and prominent nucleoli, consistent with HGSOC. This is in concordance with the review given by Lisio et al. on the heterogeneity of histopathological architecture of the subtypes of HGSOC [14]. Immunohistochemistry remains the most important diagnostic tool. Prior studies have emphasized the utility of WT1, PAX8, and CK7 with a larger panel [15]. Our case demonstrated strong diffuse nuclear PAX8 positivity, which excluded the possibility of a non-gynecologic malignancy [16] and complete absence of GFAP, which ruled out the suspicion of a primary glial tumor. The high Ki-67 index indicates aggressive mitotic activity, which is comparable to other reported metastatic cases [15].

This atypical presentation favored the possibility of delayed sanctuary-site metastasis, potentially facilitated by improved systemic disease control and prolonged survival following platinum-taxane chemotherapy. The supra-tentorial location, solid-cystic morphology, heterogeneous enhancement, marked perilesional edema, and absence of systemic recurrence at presentation further reduced initial suspicion for metastasis, highlighting the importance of maintaining a broad differential diagnosis in patients with prior ovarian cancer. Accurate distinction between metastatic carcinoma and primary glioma is critical, as management modalities differ. While gliomas typically require a chemoradiation protocol, metastatic disease is managed with surgical resection, SRS, and systemic oncologic evaluation. At four months of follow-up, the patient remains clinically stable. The absence of complete staging details and limited follow-up restrict comprehensive prognostic interpretation. 

## Conclusions

This case highlights an unusual presentation of metastatic HGSOC to the brain, with clinical and radiologic features similar to a primary temporal lobe glioblastoma in a 69-year-old female patient previously treated with surgery and adjuvant chemotherapy. Absence of common systemic symptoms with no concurrent extra-cranial disease contributed to the diagnostic dilemma. The final diagnosis was achieved only after gross total excision and histopathologic examination supplemented by immunohistochemistry. This report underscores the significance of maintaining a high index of suspicion for metastatic disease in patients with a prior history of malignancy, even after an interval of apparent complete remission, while reinforcing the crucial role of histopathology and immunoprofiling in diagnosis. It may help clinicians recognize this diagnostic pitfall to prevent misclassification and guide appropriate oncological management. While limited by short follow-up, this report contributes to existing literature on atypical presentations of intracranial metastases in ovarian carcinoma.
